# Outbreak of Human Trichinellosis — Arizona, Minnesota, and South Dakota, 2022

**DOI:** 10.15585/mmwr.mm7320a2

**Published:** 2024-05-23

**Authors:** Shama Cash-Goldwasser, Dustin Ortbahn, Muthu Narayan, Conor Fitzgerald, Keila Maldonado, James Currie, Anne Straily, Sarah Sapp, Henry S. Bishop, Billy Watson, Margaret Neja, Yvonne Qvarnstrom, David M. Berman, Sarah Y. Park, Kirk Smith, Stacy Holzbauer

**Affiliations:** ^1^Epidemic Intelligence Service, CDC; ^2^South Dakota Department of Health; ^3^University of Minnesota, Minneapolis, Minnesota; ^4^Arizona Department of Health Services; ^5^Maricopa County Department of Public Health, Phoenix, Arizona; ^6^Lakeview Clinic, Waconia, Minnesota; ^7^Division of Parasitic Diseases and Malaria, Center for Global Health, CDC; ^8^Medical Affairs, Karius, Inc., Redwood City, California; ^9^Minnesota Department of Health; ^10^Division of State and Local Readiness, Center for Preparedness and Response, CDC.

**Summary**

**What is already known about this topic?**

Human trichinellosis cases in the United States are rare and are usually acquired through consumption of wild game.

**What is added by this report?**

Among eight persons who shared a meal that included the meat of a black bear harvested in Canada and frozen for 45 days, six trichinellosis cases were identified. The meat was grilled with vegetables and served rare; two cases occurred in persons who ate only the vegetables. Motile freeze-resistant *Trichinella nativa* larvae were identified in remaining meat frozen for >15 weeks.

**What are the implications for public health practice?**

Cooking meat to an internal temperature of ≥165°F (≥74°C) is necessary to kill *Trichinella* spp. parasites. *Trichinella*-infected meat can cross-contaminate other foods, and raw meat should be kept and prepared separate from other foods to prevent cross-contamination.

## Abstract

Trichinellosis is a parasitic zoonotic disease transmitted through the consumption of meat from animals infected with *Trichinella* spp. nematodes. In North America, human trichinellosis is rare and is most commonly acquired through consumption of wild game meat. In July 2022, a hospitalized patient with suspected trichinellosis was reported to the Minnesota Department of Health. One week before symptom onset, the patient and eight other persons shared a meal that included bear meat that had been frozen for 45 days before being grilled and served rare with vegetables that had been cooked with the meat. Investigation identified six trichinellosis cases, including two in persons who consumed only the vegetables. Motile *Trichinella* larvae were found in remaining bear meat that had been frozen for >15 weeks. Molecular testing identified larvae from the bear meat as *Trichinella nativa*, a freeze-resistant species. Persons who consume meat from wild game animals should be aware that that adequate cooking is the only reliable way to kill *Trichinella* parasites and that infected meat can cross-contaminate other foods.

## Investigation and Results

### Index Patient Notification

In July 2022, the Minnesota Department of Health was notified of a man aged 29 years who was hospitalized with fever, severe myalgias, periorbital edema, eosinophilia, and other laboratory abnormalities (Table); health care providers suspected trichinellosis. The patient had sought care for his symptoms, which commenced in early July, four times and had been hospitalized twice over a 17-day period. During his second hospitalization, providers obtained a history of bear meat consumption, and empiric albendazole treatment for probable trichinellosis was initiated. An investigation was launched to confirm the diagnosis, identify additional cases, and ascertain the source of infection to prevent future cases. The index patient’s diagnosis was confirmed by a positive *Trichinella* immunoglobulin (Ig) G antibody test result.

**TABLE:** Demographic characteristics, clinical data, and laboratory test results from persons who consumed a meal that included bear meat infected with *Trichinella nativa* — Arizona, Minnesota, and South Dakota, 2022

Case status	Age, yrs, sex	Consumed bear meat	Signs and symptoms	Hospitalized	Received trichinellosis-directed treatment	WBC count, (x 1,000)/mL, (% eos)*	Creatine kinase,* units/L	*Trichinella* antibody test results	Metagenomic sequencing test results
Confirmed	12, F	Yes	Abdominal pain, myalgias, fever, and periorbital edema	Yes	Yes, albendazole	8 (37)	2,495**^†^**	Positive	Positive, *Trichinella* species
Confirmed	29, M	Yes	Abdominal pain, diarrhea, myalgias, fever, and periorbital edema	Yes	Yes, albendazole	27 (22)	1,040^§^	Positive	Positive, *Trichinella* species
Probable	29, F	No^¶^	Myalgias and fever	No	No	ND	ND	ND	ND
Probable	54, F	No^¶^	Headache and myalgias	No	No	ND	ND	Negative	ND
Probable	57, M	Yes	Diarrhea, myalgias, fever, and periorbital edema	Yes	Yes, albendazole	13 (9)	323**	Negative	ND
Probable	62, M	Yes	Diarrhea and headache	No	No	ND	ND	Negative	ND
Negative	14, M	Yes	None	NA	No	ND	ND	ND	ND
Negative	61, F	Yes	None	NA	No	ND	ND	Negative	ND

**Abbreviations:** eos = eosinophils; F = female; M = male; NA = not applicable; ND = not done; WBC = white blood cell.

* Initial results are from hospitalization during which trichinellosis was suspected. Reference ranges varied among different laboratories that conducted testing.

^†^ Reference range = 4–88.

^§^ Reference range = 39–208.

^¶^ Consumed vegetables that were cooked and served with the bear meat.

** Reference range = 39–308.

### Potential Exposure Source Identification

Six days before symptom onset in the index patient, he and eight extended family members from three states (Arizona, Minnesota, and South Dakota) had gathered for several days in South Dakota and shared a meal that included kabobs made from the meat of a black bear (*Ursus americanus*), which had been harvested by one of the family members in northern Saskatchewan, Canada in May 2022. The hunting outfitter had recommended freezing the meat to kill parasites. The meat was frozen in a household freezer* for 45 days until being thawed and grilled with vegetables. The meat was initially inadvertently served rare, reportedly because the meat was dark in color, and it was difficult for the family members to visually ascertain the level of doneness. After some of the family members began eating the meat and noticed that it was undercooked, the meat was recooked before being served again. The family reunion concluded before onset of illness in the index patient.

### Laboratory Investigation and Case Definition

Public health authorities in Arizona, Minnesota, and South Dakota interviewed eight of the nine persons who had attended the implicated meal. The ninth attendee was a person aged <18 years whose exposure status could not be confirmed; however, that person reportedly remained healthy. Testing of paired acute and convalescent sera for *Trichinella* IgG antibodies was recommended for the eight exposed persons and was completed for six. Pathogen-agnostic microbial cell-free metagenomic DNA sequencing (*1*) was performed on plasma samples from the index patient and one other person who had sought care twice before being hospitalized with fever, myalgias, abdominal pain, periorbital edema, and laboratory abnormalities. Trichinellosis cases were classified according to the 2014 case definition from the Council for State and Territorial Epidemiologists (CSTE),^†^ (i.e., the presence of clinically compatible symptoms in a person who had consumed an epidemiologically implicated meal or meat in which the parasite was demonstrated [probable] or had a positive serologic test result for *Trichinella* antibodies [confirmed]). Samples of frozen bear meat were obtained from the household freezer and sent to CDC for artificial tissue digestion and microscopic examination for larvae and molecular testing for *Trichinella* spp.

### Additional Case Detection and Exposure Source Confirmation

Among the eight interviewed persons, five consumed the bear meat, and eight consumed the vegetables that had been cooked with it. Six of the eight persons who attended the meal, including four who consumed the bear meat and the vegetables, and two who consumed only the vegetables (but no meat), had symptoms consistent with trichinellosis, and met case criteria (two confirmed and four probable). Patients with trichinellosis ranged in age from 12 to 62 years and lived in three states: Arizona (one), Minnesota (four), and South Dakota (one). All cases were diagnosed in the patients’ state of residence. Three of the six symptomatic persons, two of whom sought care at least twice before being offered treatment, were hospitalized. The three hospitalized persons received trichinellosis-directed treatment with albendazole.^§^ All six symptomatic persons recovered; the nonhospitalized patients did not receive trichinellosis-directed treatment because their symptoms had resolved with supportive care only, and the benefit of treatment after larval invasion of muscle is unclear (*2*). Six persons submitted a serum sample, each collected within 4 weeks of symptom onset; two specimens tested positive for *Trichinella* IgG antibodies by enzyme-linked immunosorbent assay. Two persons submitted a plasma sample for microbial cell-free DNA sequencing during hospitalization for trichinellosis-compatible symptoms, and both plasma samples tested positive for *Trichinella* spp. DNA. Microscopy identified motile *Trichinella* larvae (>800 larvae/g) in samples of bear meat that had been frozen for 110 days in a household freezer (Figure). Real-time multiplex polymerase chain reaction testing (*3*) of the bear meat was positive for *T. nativa* and whole genome sequencing identified mitochondrial sequences 100% identical to *T. nativa.*

**FIGURE:**
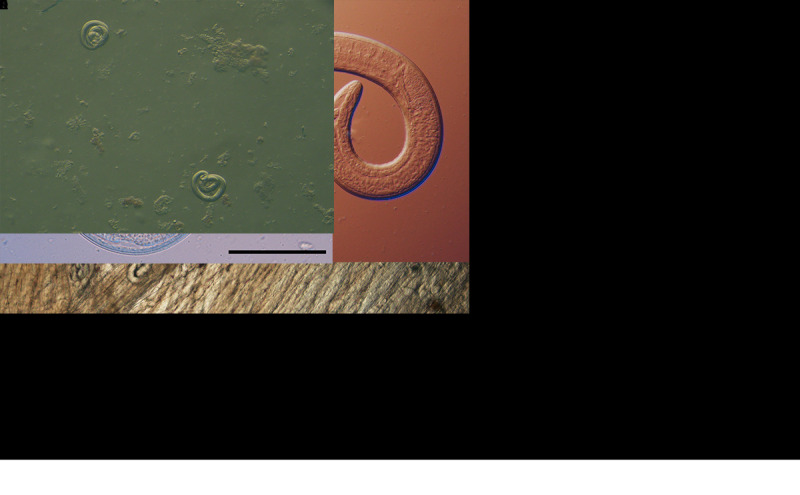
Microscopic examination of encapsulated larvae in a direct black bear meat muscle squash prep (A), larvae liberated from artificially digested bear meat (B), and motile larvae viewed with differential interference contrast microscopy (C and D)* from black bear meat suspected as the source of an outbreak of human *Trichinella nativa* infections — Arizona, Minnesota, and South Dakota, 2022 Photos/Division of Parasitic Diseases and Malaria, Center for Global Health, CDC * Scale bars = 100 *µ*m.

## Public Health Response

The family member who harvested the bear and provided meat samples for testing was advised to discard any remaining meat. All identified trichinellosis cases were reported to appropriate state health departments and to CDC. CDC notified the Public Health Agency of Canada of the outbreak and the confirmed source of infection. This activity was reviewed by CDC, deemed not research, and was conducted consistent with applicable federal law and CDC policy.^¶^

## Discussion

Trichinellosis is rarely reported in the United States. As a result of changes in pork production practices from historical norms that fostered transmission, most cases reported in recent years are attributed to consumption of meat from wild game (*4*). During January 2016–December 2022, seven U.S. trichinellosis outbreaks, including 35 probable and confirmed cases, were reported to CDC; bear meat was the suspected or confirmed source of infection in the majority of those outbreaks (CDC, unpublished data, 2022). Estimates of *Trichinella* infection prevalence among wild animal host species vary widely. A *Trichinella* infection prevalence range of at least 1% to 24% among black bears in Canada and Alaska has been reported, and even higher prevalences of *Trichinella* infection are reported among species of predators that are strict carnivores (e.g., polar bear, wolverine, and cougar) (*5*). The frequency with which black bear meat is the implicated source of human infection might be driven by hunting practices, ecological factors, and the relatively high parasite density observed in the muscle of infected black bears compared with that of other species (*6**,**7*).

Because symptoms of trichinellosis are typically nonspecific, diagnosis of infection requires a high index of suspicion; however, periorbital edema and certain laboratory abnormalities (e.g., eosinophilia and elevated creatine kinase levels) can provide etiologic clues. In this outbreak, two of the hospitalized patients sought care multiple times before receiving a diagnosis. Four of the six patients met clinical and epidemiologic criteria and thus were considered probable cases. Laboratory confirmation can be challenging because of the limited sensitivity of antibody testing early in illness (*8*); in this investigation, acute *Trichinella* IgG test results were positive in only two of six tested patient specimens. The clinical utility of trichinellosis test results obtained after acute illness is limited, and historically, public health investigators have had difficulty obtaining convalescent serum samples from persons who have recovered. Laboratory criteria in the current CSTE trichinellosis case definition do not include nucleic acid testing of human specimens. The sensitivity of such assays to detect *Trichinella* DNA in blood is uncharacterized; however, plasma samples from both patients tested by metagenomic sequencing (*1*) yielded positive results for *Trichinella* DNA. As demonstrated in this outbreak, pathogen-agnostic molecular assays can be useful for detection of rare diseases when standard workup is unrevealing and if other diagnostic tests lack sensitivity.

## Implications for Public Health Practice

Although freezing kills *Trichinella* species commonly implicated in pork-associated outbreaks, freeze-resistant *Trichinella* species, including *T. nativa* and the T6 genotype (*9*), predominate in Arctic and sub-Arctic regions (*6*). Larval motility was observed in bear meat that had been frozen for nearly 4 months (110 days). Persons who consume game meat, especially that harvested in northern latitudes, should be informed that adequate cooking is the only reliable way to kill *Trichinella* parasites. Cooking wild game meat to an internal temperature of ≥165°F (≥74°C) is recommended by public health authorities**; temperatures should be verified with a meat thermometer. As demonstrated in this outbreak, the color of meat is not a good indicator of cooking adequacy. Safe handling of raw meat (i.e., separating raw or undercooked meat and its juices from other foods) is recommended to prevent trichinellosis; this investigation and previous investigations suggest that *Trichinella*-infected meat can cross-contaminate other foods (*10*). Government and private entities that oversee and organize hunting should educate hunters about these risks and effective preventative measures.

## Footnotes

* The temperature of the freezer is not known.

†
https://ndc.services.cdc.gov/case-definitions/trichinellosis-2014/

¶ 45 C.F.R. part 46, 21 C.F.R. part 56; 42 U.S.C. Sect. 241(d); 5 U.S.C. Sect. 552a; 44 U.S.C. Sect. 3501 et seq.

**
https://www.cdc.gov/trichinellosis/prevention/index.html
